# MicroRNA-222 influences migration and invasion through MIA3 in colorectal cancer

**DOI:** 10.1186/s12935-017-0447-1

**Published:** 2017-08-29

**Authors:** Heli Gao, Xuejing Cong, Jianfeng Zhou, Mei Guan

**Affiliations:** Department of Oncology, Peking Union Medical College Hospital, Chinese Academy of Medical Sciences and Peking Union Medical College, Beijing, People’s Republic of China

**Keywords:** miR-222, MIA3, Colorectal cancer, Migration, Invasion

## Abstract

**Background:**

miR-222 has been reported to be overexpressed in colorectal cancer and it influences cancer cell proliferation, drug resistance and metastasis. However, the underlying molecular mechanism of miR-222 in colorectal cancer cell invasion and migration has not been thoroughly elucidated to date.

**Methods:**

The cell cycle distribution and apoptosis were assessed by flow cytometry. Cell migration and invasion were analyzed by Transwell assays. The possible target gene of miR-222 was searched and identified by bioinformatics, dual luciferase reporter assay and western blot analysis. The siRNA method was used to confirm the function of the target gene.

**Results:**

Overexpression of miR-222 effectively promotes migration and invasion of colorectal cancer (CRC) cells in vitro. Bioinformatics and the dual luciferase reporter assay revealed that miR-222 specifically targeted the 3′-UTR of melanoma inhibitory activity member 3 (MIA3), down-regulating its expression at the protein level. Inhibition of MIA3 by siRNA enhanced the migration and invasion of CRC cell lines.

**Conclusions:**

Our study showed that miR-222 enhances the migration and invasion in CRC cells, primarily by down-regulation of MIA3.

**Electronic supplementary material:**

The online version of this article (doi:10.1186/s12935-017-0447-1) contains supplementary material, which is available to authorized users.

## Background

Colorectal cancer (CRC) is the one of the most common malignancies worldwide with an unfavorable overall survival rate [1]. The poor prognosis and increasing incidence of CRC highlights the need to reveal the pathological mechanisms governing CRC formation and progression.

MicroRNAs (miRNAs) are a class of small non-coding, single-stranded RNAs that play an important role in many cellular processes, such as tumorigenesis [2] and immune defense [3]. Recent evidence has suggested that miRNAs could function as oncogenes or tumor suppressors [4, 5]. As a new biomarker, miRNAs are valuable in tumor diagnosis and treatment.

Aberrant expression of miRNAs has been reported in a variety of human tumors, including CRC [6]. For example, miR-21 is highly expressed in advanced CRC tissues, which is associated with CRC cell-cycle arrest and worse survival [7, 8]. miR-143 and miR-145 (miR-143/145) are down-regulated in CRC tissue, and they act as tumor suppressors [9, 10].

Overexpression of miR-222 has been reported in the plasma and tissues of CRC patients [11, 12]. Our previous study showed that the miR-222 expression level is significantly decreased in CRC cell lines after treatment with 5-fluorouracil (5-FU) or oxaliplatin (L-OHP) [13]. The dysregulation of miR-222 could affect CRC cell proliferation and multidrug resistance. Tsunoda et al. [14] found that KRAS regulates miR-222 in CRC cells. Xu et al. [15] found that miR-222 modulates multidrug resistance in CRC by down-regulating ADAM-17. Liu et al. [16] showed that miR-222 promotes CRC cell proliferation by acting in a positive feedback loop to increase the expression levels of RelA and STAT3. These studies underscore the need for an in-depth search of miR-222 functions in CRC cells and underlying mechanisms. In this study, we observed thatmiR-222 enhanced migration and invasion through MIA3 in colorectal cancer.

## Methods and materials

### Tumor cell culture

Human colorectal adenocarcinoma cell lines, HCT8 and Lovo, were purchased from Cell Resource Center, IBMS, CAMS/PUMC (Beijing, China). All cell lines were maintained in DMEM medium (Gibco, Paisley, UK) supplemented with 10% fetal calf serum (FCS, Gibco, Paisley, UK) in humidified 5% CO_2_/95% atmosphere at 37 °C.

### miRNA and siRNA transfection

The miRNA and siRNA transfection method was performed as previously described [17]. The synthetic miR-222 mimic (forward, 5′-AGC UAC AUC UGG CUA CUG GGU-3′ and reverse, 5′-CCA GUA GCC AGA UGU AGC UUU-3′), miR-222 inhibitor (5′-ACC CAG UAG CCA GAU GUA GCU-3′), mimic control (forward, 5′-UUC UCC GAA CGU GUC ACG UTT-3′ and reverse,5′-ACG UGA CAC GUU CGG AGA ATT-3′) and inhibitor control (5′-CAG UAC UUU UGU GUA GUA CAA-3′) were purchased from GenePharma (GenePharma Inc., Shanghai, China). The siRNAs for MIA3 were synthesized (Invitrogen Inc.) and the sequences of si-MIA3 are 5′-CCA GGU AGU UCA UGA AUA UTT-3′ (MIA3-siRNA-1), 5′-CGC AGA ACA UCA CAU UAA ATT-3′ (MIA3-siRNA-2) and 5′-CGG ACA CAG ACU GCA AUA UTT-3′ (MIA3-siRNA-3).

### RNA reverse transcription and qRT-PCR

Total RNA was extracted using the Trizol total RNA isolation reagent (Invitrogen) and purified with the Column DNA Erasol kit (TIANGEN, Beijing, China) according to the manufacturers’ instructions. The mRNA levels were assessed with qRT-PCR using SYBR Green I (TaKaRa, Dalian, China). The gene expression level was normalized to an endogenous reference gene, GAPDH. The experiments were performed in triplicate. The primers for GAPDH are Forward 5′-GAA GGT GAA GGT CGG AGTC-3′ and Reverse 5′-AAG ATG GTG ATG GGA TTTC-3′. The primers for MIA3 are Forward 5′-AAGTTCCAACAGATGAGACGGA-3′ and Reverse 5′-GGTTCAGGTTCCCTTTCCTTAG-3′. The primers for miR-222 and U6 were purchased from QIAGEN, the sequences of hsa-miR-222 and U6 are 5′-AGC TAC ATC TGG CTA CTG GGT-3′ and 5′-CAA GGA TGA CAC GCA AAT TCG-3′, respectively. Reverse transcription of miRNAs was performed with a miScript Reverse Transcription Kit (QIAGEN, Duesseldorf, Germany). The expression of mature miRNAs was determined using miRNA-specific quantitative qRT-PCR (TaKaRa, Dalian, China). The expression levels were normalized to the U6 endogenous control and measured by the comparative Ct (∆∆Ct) method.

### Western blot analysis

After washing twice with PBS, cells were lysed in ice-cold Radio Immunoprecipitation Assay (RIPA) lysis buffer (Beyotime, Nanjing, China) and manually scraped from culture plates. Proteins were separated on 10% sodium dodecyl sulfate-polyacrylamide gel electrophoresis (SDS-PAGE) gels, electro blotted onto a polyvinylidenedifluoride (PVDF) membrane, and incubated with anti-MIA3 antibody (1/1000; GeneTex, CA) or anti-GAPDH antibody (1/2000; Santa Cruz Biotechnology, CA). Then, samples were incubated with a secondary anti-rabbit or anti-mouse horseradish peroxidase-conjugated antibody (1/3000; Santa Cruz Biotechnology, Santa Cruz, CA). Antibody-antigen complexes were detected using a chemiluminescent ECL reagent (Millipore).

### In vitro migration and invasion assay

Transwell chambers (8-μM pore size; Costar) were used in the in vitro migration assay. HCT8 and Lovo cells were transfected with the miR-222 mimic, mimic control, miR-222 inhibitor, inhibitor control, Si-MIA3 and SiRNA control. After 48 h, cells were detached with trypsin, washed with PBS and resuspended in serum-free medium. Since HCT8 and Lovo cells have different migration and invasion capacities, the Transwell assay differs slightly among them. For HCT8 cells, the seeding cell number was 2 × 10^5^ (mimic control and miR-222 mimic group) and 4 × 10^5^ (inhibitor control and miR-222 inhibitor). For Lovo, the seeding cell number was 5 × 10^4^ (mimic control and miR-222 mimic group) and 1 × 10^5^ (inhibitor control and miR-222 inhibitor). The number of selected cells is convenient for counting under a microscope. The time for the migration assay was 12 h and that for the invasion assay was 36 h. The cells that had not migrated were removed from the upper surfaces of the filters using cotton swabs, and the cells that had migrated to the lower surfaces of the filters were fixed with 4% paraformaldehyde solution and stained with crystal violet. Images of three random fields (10× magnification) were captured from each membrane, and the number of migratory cells was counted. Similar inserts coated with Matrigel were used to determine the invasive potential.

Since manual counts may have errors, we have added another detected method for migration and invasion. Serum starved cells (2 × 10^5^ cells) were plated over Transwell inserts in the migration assay. In the invasion assay, the Transwell inserts were pre-coated with growth-factor reduced matrigel and were permitted to invade towards serum-contained in the bottom chamber for 12 (migration assay) or 36 (invasion assay) hours. Non-migrated cells were swabbed from the tops of half of the inserts (‘samples’, containing only invaded cells) and retained in the others (‘controls’, all cells). Inserts were fixed with 4% paraformaldehyde solution and stained for 10 min with crystal violet and washed with water. Membranes were destained in 10% acetic acid and absorbance was read at 570 nm. The percent of migration and invasion was calculated as absorbance of samples/absorbance of controls × 100 [18].

### In vitro cell cycle assay

HCT8 cells transfected with the miR-222 mimic, mimic control, miR-222 inhibitor and inhibitor control were cultured for 3 days and then harvested and quantified. Cells were collected by trypsin treatment and counted with a Cell counter. A total of 500,000 cells per well were fixed, permeabilized and stained in accordance with the manufacturer’s instructions. The sample was analyzed by flow cytometry using a COULTER EPICS XL. Data were analyzed using MultiCycle software to generate the percentages of cells in the G1, S and G2 to Mphases of the cell cycle.

### Apoptosis assay

HCT8 cells transfected with the miR-222 mimic, mimic control, miR-222 inhibitor and inhibitor control were cultured for 3 days and then harvested and quantified. They were stained with an Annexin-V kit (BD, USA) using standard procedures and analyzed by flow cytometry (FACS Vantage). Annexin-V was evaluated with FL1 channel and PI was evaluated with FL2 channel. Ten thousand events were analyzed with a flow cytometer BD Accuri™ C6 (BD Biosciences).Data were analyzed with CFlow plus 1.0.

### Dual luciferase reporter gene construct and dual luciferase reporter assay

A fragment of the MIA3 (Thymidylate synthase) 3′UTR containing the predicted binding site for hsa-miR-222, and the flanking sequence on each side was synthesized with a short extension containing cleavage sites for *Xba*I (5′ end) and NotI (3′ end) (Additional file 1: Table S1). A second fragment containing a mutated sequence of the binding site was also synthesized. The two constructs were termed WT (Gene-wild type) and MT (Gene-mutant). The fragments were cloned into the psiCHECK™-2 vector (Promega Corporation, Madison, WI). Then, 10 ng of WT,MT and control vectors and 200 nmol/L miR-222 mimic were transfected into 293T cells using Lipofectamine 2000 (Invitrogen, Carlsbad, CA) according to the manufacturer’s instructions. Cells were harvested 24 h after transfection and assayed for renilla and firefly luciferase activity using the Dual-Luciferase Reporter Assay System (Promega, Madison, WI, USA).

#### Clinical specimens

Thirty-nine paraffin embedded CRC tumor tissues were obtained from patients with CRC treated at Peking Union Medical College Hospital (PUMCH) between 2005 and 2010. All clinical specimens were obtained under approval of the institutional Ethics Committee. Informed consent was obtained from each subject. The histology of cancer tissues was determined by a pathologist.

MIA3 expression was evaluated by immunohistochemistry staining. Briefly, after 5-μm sections were deparaffinized, antigen retrieval was performed with heat-induced epitope retrieval and 10 mM citrate buffer. Sections were incubated with a monoclonal antibody against MIA3 (Abcam, UK) at 1:100 dilution. The MIA3 antibody was detected using the avidin–biotin–peroxidase technique (DakoLSAB Kit, Dako). The expression levels of MIA3 were determined by a pathologist. The classification of “−, +” was defined by the percentage of MIA3 positive cells at the levels of <10 and 10–100%, respectively.

### Statistical analysis

The data are presented as the mean ± standard deviation. An analysis of variance was used to evaluate the data from the apoptosis assays. Comparisons between groups were analyzed using t-tests (two-sided) with SPSS 19.0. Differences with P values less than 0.05 are considered significant. The correlation between miR-222 and MIA3 expression was determined by the SPSS assay (Pearson assay).The Kaplan–Meier method was used to analyze the disease-free survival (DFS) of the patients.

## Results

### miR-222 promotes invasion and migration of CRC cell lines in vitro

**Fig. 1:**
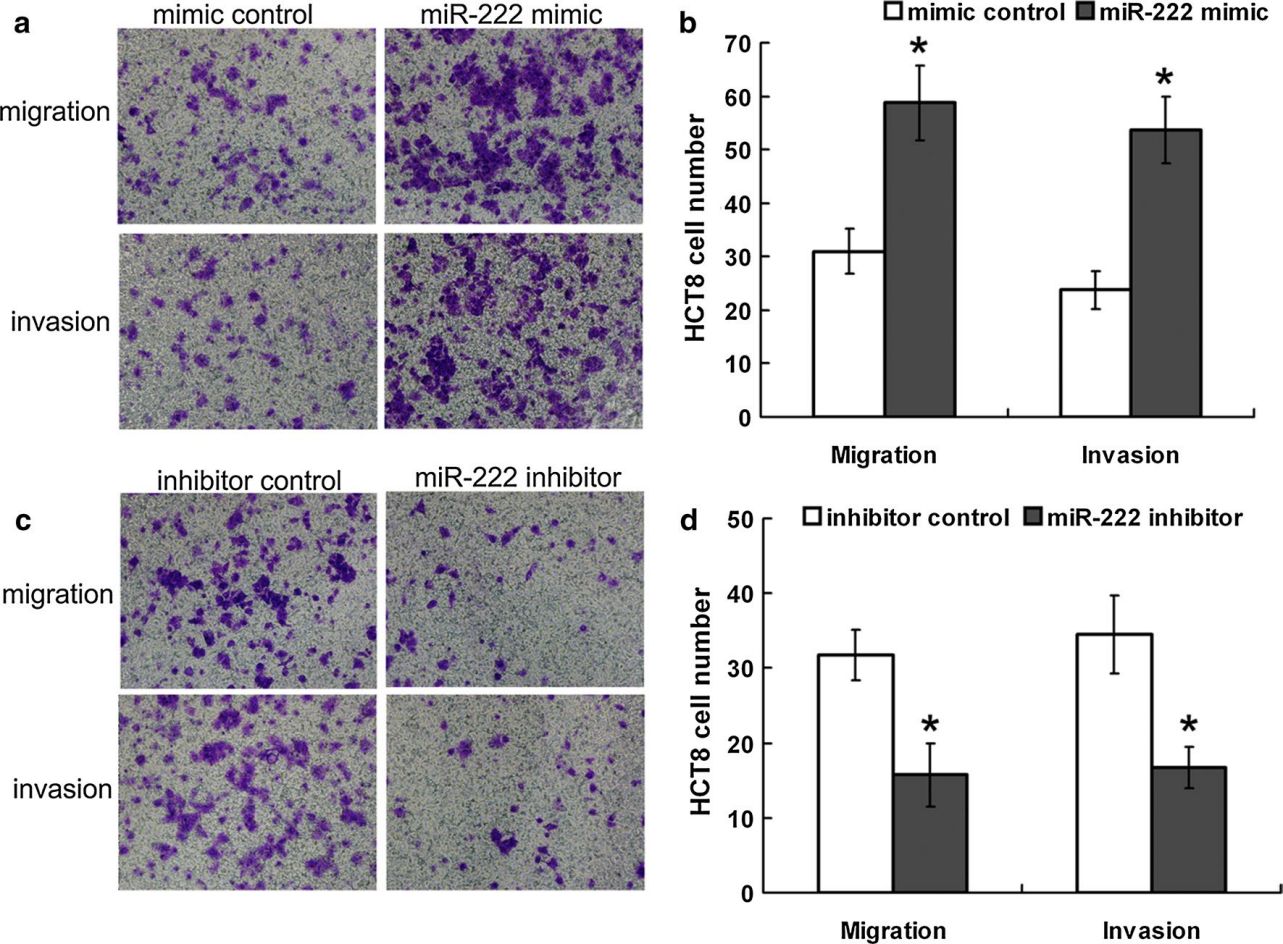
miR-222influence on migration and invasion of HCT8 cells. **a**, **b** Transwell migration (n = 4) and invasion (n = 4) assays showed that HCT8 cells transfected with the miR-222 mimics (800 nM) had higher invasive and migratory potentials than the control (mimics control). **a** A microscopic image of crystal violet staining. **b** The statistical results. **c**, **d** Transwell migration (n = 4) and invasion (n = 4) assays showed that HCT8 cells transfected with the miR-222 inhibitor (800 nM) had lower invasive and migratory potentials than the control (inhibitor control). **c** A microscopic image of crystal violet staining. **d** The statistical results. Data represent the mean ± SD of four independent experiments. *P < 0.05

We first investigated the effect of miR-222 on the invasion and migration of CRC. Two well-established CRC cell lines, HCT8 and Lovo, were utilized. The HCT8 cell line was transfected with miR-222 mimic, mimic control, miR-222 inhibitor and inhibitor control. The transfection efficiency in HCT8 cells is shown in Additional file 2: Figure S1. HCT8 cells were subjected to cell invasion and migration assays by the Transwell chambers assay. Compared to the control group, HCT8 cells transfected with miR-222 mimic have significantly higher invasion and migration capacity, while CRC cells transfected with miR-222 inhibitor have dramatically lower invasion and migration capacity (Fig. 1; Additional file 4: Figure S3). Similar results were obtained in Lovo cells (Additional file 3: Figure S2, Additional file 4: Figure S3). These results suggested that miR-222 promoted invasion and migration of CRC.

### miR-222 does not influence cell cycle or apoptosis of CRC cell lines in vitro

**Fig. 2:**
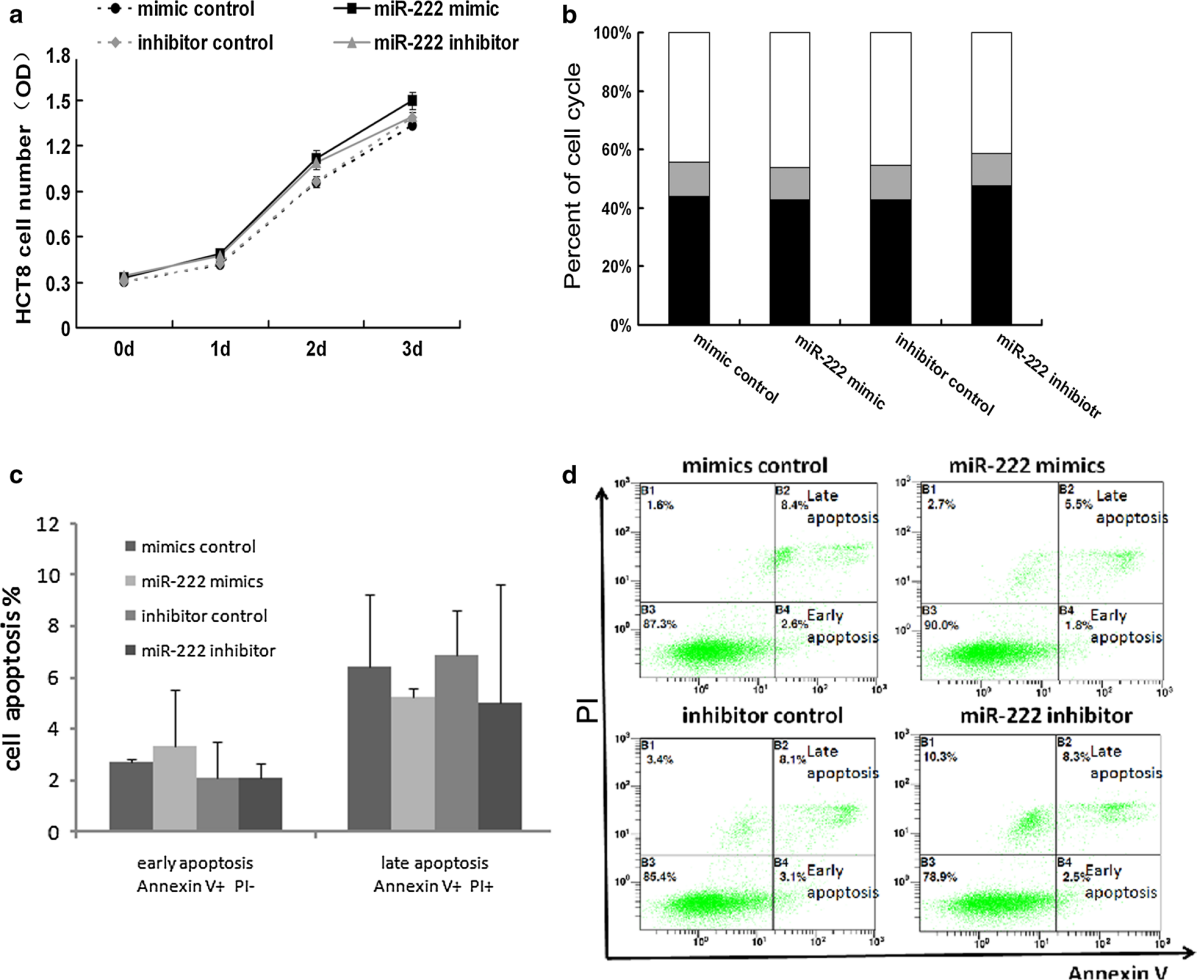
miR-222 had no effect on HCT8 cell proliferation, cell cycle and apoptosis. HCT8 cells were transfected with miR-222 mimic, mimic control, miR-222 inhibitor or inhibitor control. **a** Cell proliferation detected by Cell Titer 96^®^ A Queous Non-Radioactive Cell Proliferation Assay (MTS).Cell numbers were determined by MTS after 0, 1, 2 and 3 days. Data represent the mean ± SD of 6 independent experiments. **b** The cells were stained with PI and detected by FACS. The cell cycle was compared among the G0/G1 (*black bar*), G2/M (*gray bar*) and S stages (*white bar*). The data represent the mean ± SD, which are further detailed in Additional file 1: Table S1. **c**, **d** Apoptosis was detected by FACS. HCT8 cells were marked with Annexin V and PI, Annexin V+/PI− and Annexin V+/PI+ indicating early and late apoptosis, respectively. Data represent the mean ± SD of six independent experiments

We then tested the potential role of miR-222 in CRC cell growth. HCT8 cells were transfected with miR-222 mimic, mimic control, miR-222 inhibitor or inhibitor control. Neither over-expression nor down-regulation of miR-222 obviously influenced the CRC cell proliferation and cell cycle (Fig. 2a, b; Additional file 1: Table S2).We evaluated CRC cell apoptosis through flow cytometry. HCT8 cells were marked with Annexin V and PI. Annexin V+/PI− and Annexin V+/PI+ represented early and late apoptosis, respectively. No statistically significant difference was found in apoptosis among each group (Fig. 2c, d).

### miR-222 directly increased MIA3 expression by interacting with its 3′UTR

**Fig. 3:**
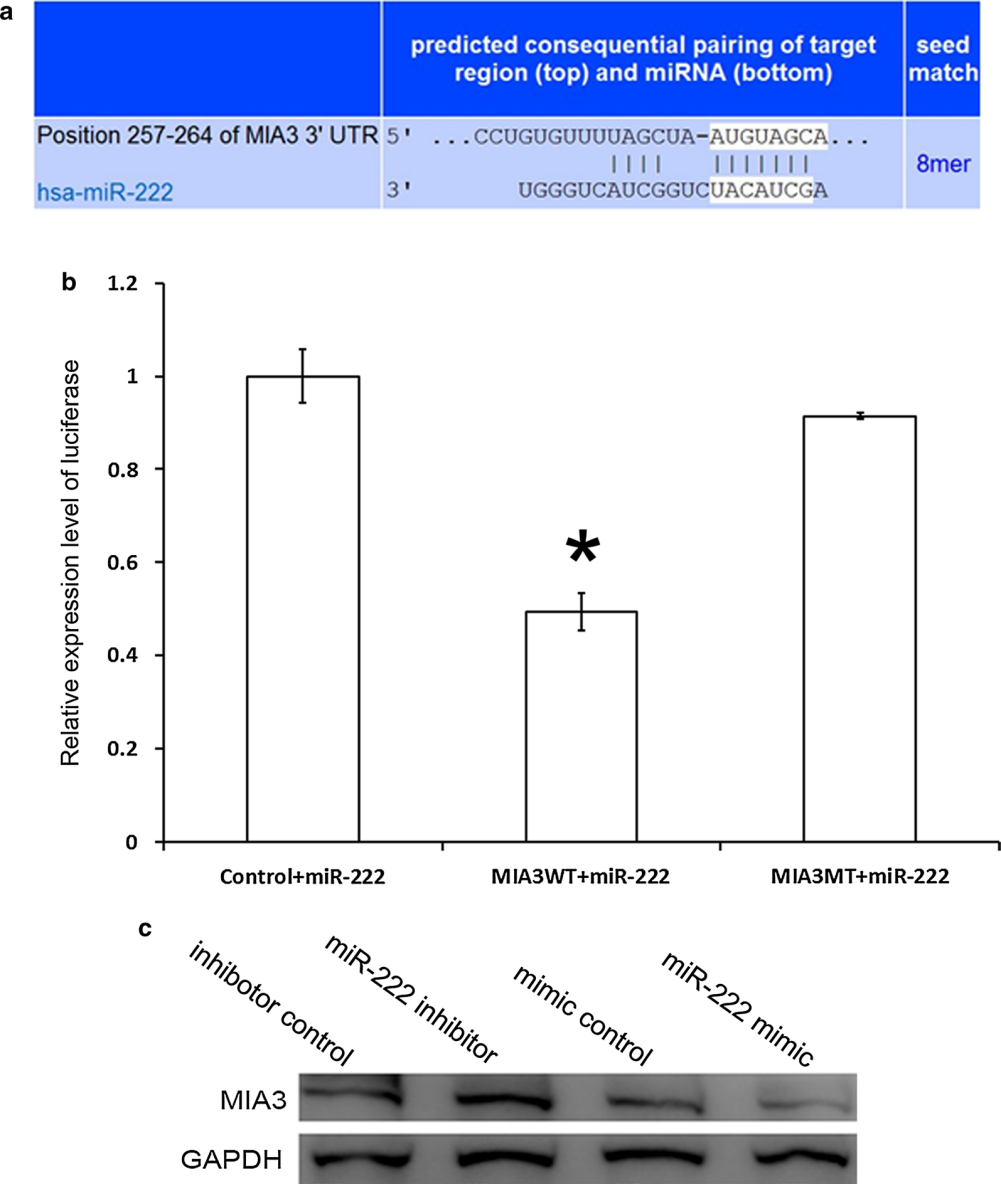
MIA3 was a direct target of miR-222. **a** The putative binding site for miR-222 in the 3′UTR of MIA3 was revealed by TargetScan. **b** The miR-222 binding site on MIA3 3′UTR was confirmed by the luciferase assay in 293T cells after cotransfection with a plasmid containing a fragment of MIA3 3′UTR that included either the wild-type or mutant predicted miR-222 binding site. Data represent the mean ± SD of at least three independent experiments. *P < 0.01. Control is the plasmid without sequence. *WT* wild type of MIA3 3′UTR, *MT* mutation type of MIA3 3′UTR. **c** Western blot assay of MIA3 protein levels in HCT8 cells treated with miR-222 mimics, mimics control, miR-222 inhibitor and inhibitor control (800 nM)

miRNAs exert their effect by regulating target genes at the post-transcriptional level. To identify a potential target of miR-222, we first used TargetScan and PicTar web to predict target genes. Then, we searched the literature for the reported functions of these genes. We are particularly interested in MIA3, which has been shown to be down-regulated or even lost in colon cancer, and it influences the migration and invasion of CRC cells [19]. Figure 3a demonstrated the conserved binding site of miR-222 in the MIA3 3′UTR. Then, we used dual luciferase reporter assays to confirm whether MIA3 is a direct target of miR-222. The renilla luciferase activity in MIA3-WT-transfected cells decreased by more than 25% in miR-222 mimic-cotransfected cells compared to mimic control-cotransfected cells and the MIA3-MU-transfected cells (Fig. 3b). Western Blot results further showed decreased MIA3 expression after transfection with the miR-222 mimic (Fig. 3c).

### Down-regulation of MIA3 increases the migration and invasion of CRC cell lines

**Fig. 4:**
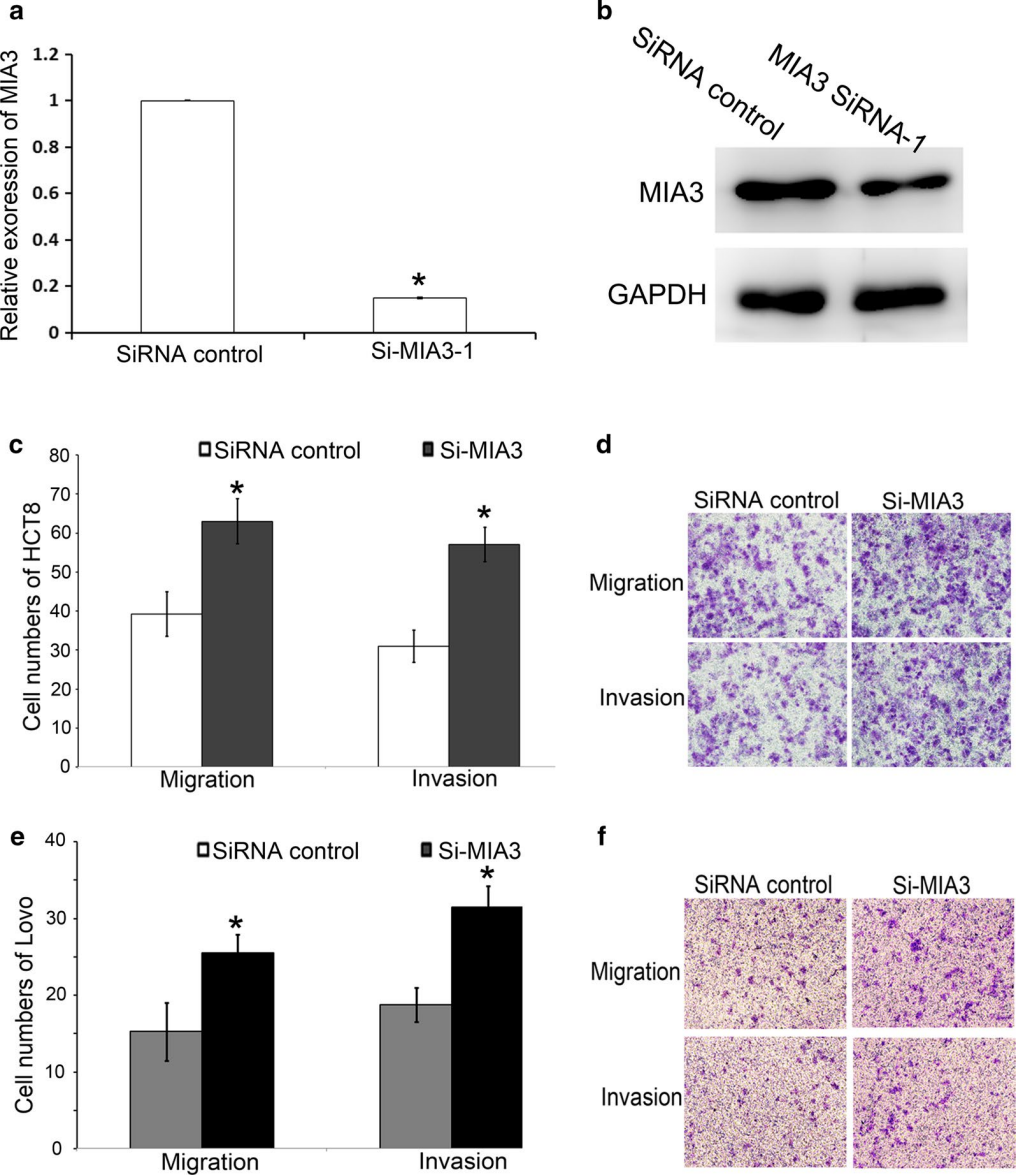
MIA3 regulated HCT8 and Lovo cell migration and invasion. **a** The interfering effect of MIA3-siRNA-1 with RT-PCR analysis. **b** Western blot assay showed decreased MIA3 expression after transfection with the MIA3-siRNA-1 (200 nM). Transwell migration (n = 4) and invasion (n = 4) assays showed that HCT8 cells (**c**, **d**) and Lovo cells (**e**, **f**) that were transfected with the MIA3-siRNA-1 (200 nM) had greater invasive and migratory potentials than the control (siRNA control). **c**, **e** The statistical results,*P < 0.01. **d**, **f** A microscopic image of crystal violet staining. Data represent the mean ± SD of four independent experiments

Then, we evaluated whether down-regulation of the MIA3 levels could affect CRC cell migration and invasion. Three small interfering RNAs (siRNAs) were designed to target MIA3 and the interference effect of MIA3-siRNA-1 is significant (mRNA expression was detected by RT-PCR, Fig. 4a; protein expression was detected by Western blot, Fig. 4b). Therefore, we chose MIA3-siRNA-1 for further study. The cell migration and invasion assay results showed that after down-regulation of MIA3, HCT8 cell migration and invasion were significantly increased compared with the control group (Fig. 4c, d; Additional file 5: Figure S4A). Similar results were obtained in Lovo cell lines (Fig. 4e, f; Additional file 5: Figure S4B).

### Correlation between miR-222 and MIA3 expression in CRC cell lines and colon cancer patients

We detected the expression of MIA3 (Western blot) and miR-222 (RT-PCR) in 4 CRC cell lines. The expression level of MIA3 in HCT8 and SW480 cells was higher than in HCT116 and Lovo cells, but the expression level of miR-222 was lower than HCT116 and Lovo cells. However, these data are insufficient to calculate the correlation between MIA3 and miR-222 (Additional file 6: Figure S5).

**Fig. 5:**
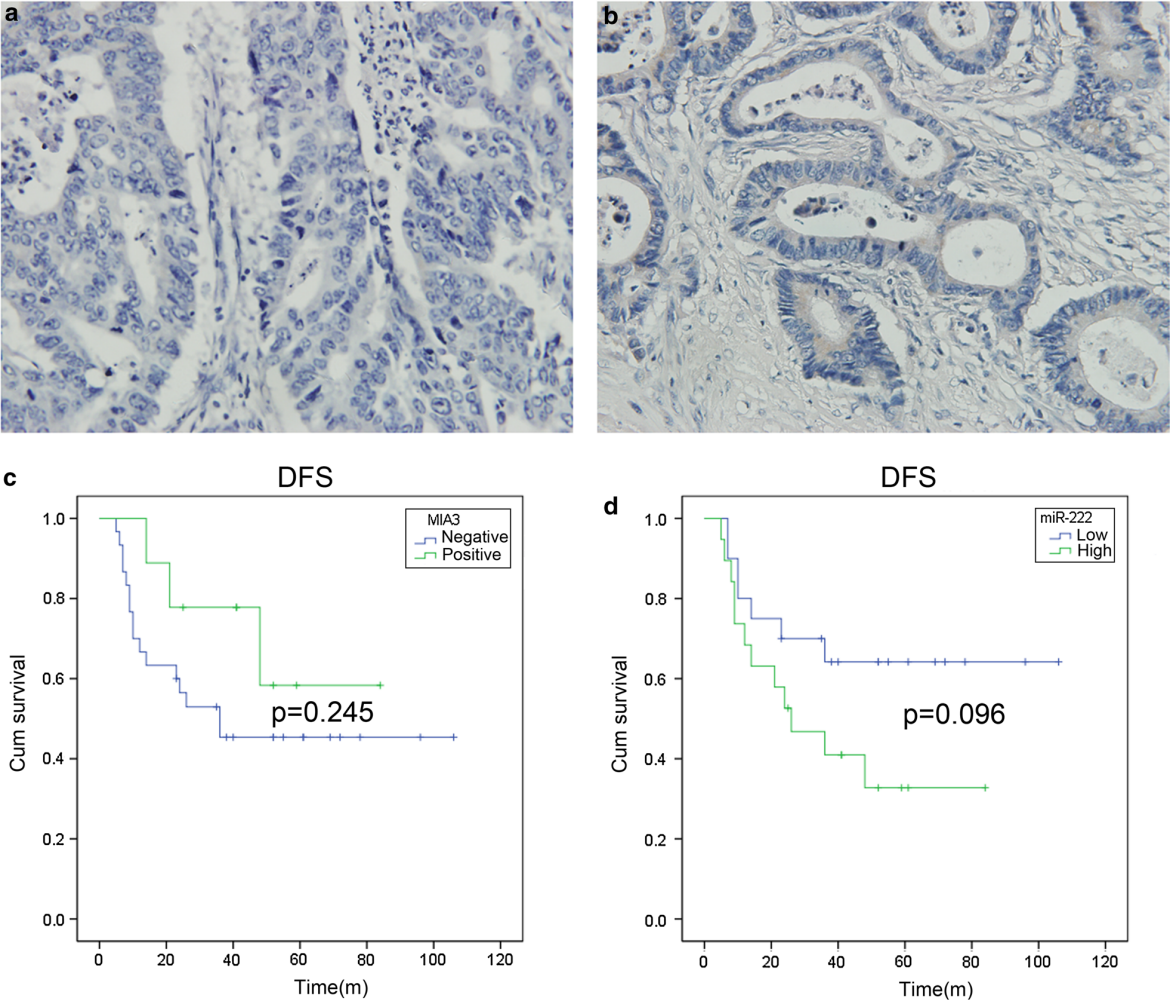
Influence of different factors affecting the DFS of the patients. **a**, **b** Immunohistochemistry of MIA3 in colorectal cancer. **a** Negative and **b** positive. **c** The influence of MIA3 expression on the DFS of CRC patients (P = 0.289). **d** The influence of miR-222 expression on the DFS ofCRC patients (P = 0.074)

We obtained clinical samples from 39 CRC patients. Additional file 1: Table S3 showed the clinical characteristics and expression of miR-222 and MIA3 in these patients. To investigate the correlation between miR-222 and MIA3 expression, we analyzed the colon cancer tissue samples by RT-PCR and immunohistochemistry for miR-222 and MIA3, respectively. Figure 5a, b showed the characteristic expression of MIA3. The results demonstrated that the expression of miR-222 (ΔCT) was correlated with MIA3; the correlation coefficient was 0.510, which was statistically significant (P < 0.001, Additional file 1: Table S4).The higher the CT value, the lower the expression of miR-222 such that the expression levels of MIA3 and miR-222 were negatively correlated.

### Correlation between miR-222 and MIA3 expression and disease free survival (DFS) of the patients

From 2005 to 2010, patients with CRC underwent surgery in Peking Union Medical College hospital; among them, only 39 adenocarcinoma patients had follow-up data and for statistical analysis. miR-222 of CRC patients’ cancer tissues were evaluated by RT-PCR; the average of ΔCT is 4.59. Patients with high miR-222 (ΔCT < 4.59) expression had a DFS of 40.80 ± 7.64 m (25.77–55.72 m), while patients with low miR-222 (ΔCT > 4.59) expression had a DFS of 73.66 ± 9.88 m (54.29–93.03 m) (P = 0.096, Fig. 5d). Patients who were MIA3 positive had a DFS of 62.22 ± 10.03 m (42.57–81.88 m), while patients who were MIA3 negative had a DFS of 56.55 ± 8.46 m (40.00–73.17 m) (P = 0.245, Fig. 5c). The data were not statistically significant, which was probably due to the limited sample size.

## Discussion

miR-222 is considered an oncogenic gene in most epithelial tumors [20], promoting oncogenic process by targeting PTEN and TIMP3 in NSCLC and hepatocellular carcinoma [21, 22].Up-regulation of miR–222 induces an enhancement of proliferation by down-regulating its target P27^Kip1^ in ovarian and hepatocellular cancer [23, 24].

Liu et al. [16] showed that miR-222 promotes CRC cell proliferation. Therefore, we further analyzed the role of miR-222 in the cell cycle, apoptosis, invasion and migration in CRC cell lines. We found that inhibition of miR-222 significantly reduces the migration and invasion of CRC cells in vitro. microRNA performs its function by binding to the 3′UTR of target genes. Through bioinformatics and a dual-luciferase reporter assay, we found that miR-222 directly binds to the 3′UTR of MIA3. Additionally, miR-222 expression is negatively correlated with MIA3 at the protein level.

MIA3, also known as TANGO1 (transport and Golgi organization genes), is an endoplasmic reticulum resident transmembrane protein [25]. It has been demonstrated to be a tumor suppressor of malignant melanoma [26]. A recent study showed that MIA3 is down-regulated or even lost in colon cancer and that its overexpression decreases the migration and invasion of CRC cells [19]. However, the mechanism of MIA3 regulation in cancer is unclear.

Here, we analyzed the inhibitory effects induced by siRNA-mediated knockdown of MIA3, and the results demonstrated that MIA3 acts as a suppressor. Inhibiting MIA3 caused up-regulation of invasion and migration in CRC cell lines. The mechanism by which MIA3 influences the migration and invasion of colorectal cancer cells requires further study.

## Conclusions

This study’s results indicate that miR-222 mostly enhances migration and invasion in CRC cells by down-regulation of MIA3. Further studies are needed to determine the molecular mechanisms of MIA3 and the clinical value of miR-222 in CRC.

## Additional files

**Additional file 1: Table S1.** The sequence of target gene (MIA33’UTR) and mutation target gene. **Table S2.** miR-222 had no effect on HCT8 cell cycle. **Table S3.** The condition of CRC patients. **Table S4.** The correction between miR-222 and MIA3.

**Additional file 2: Figure S1.** The transfection efficiency of miR-222 inhibitor and miR-222 mimics in HCT8.

**Additional file 3: Figure S2.** miR-222 influence on migration and invasion of Lovo. (A and B) Transwell migration (n=4) and invasion (n=4) assays showing that Lovo cells transfected with the miR-222 mimics (800 nM) had higher invasive and migratory potentials than the control (mimics control). (A) The statistical results,*P<0.05. (B) A microscopic image of crystal violet staining. (C and D) Transwell migration (n=4) and invasion (n=4) assays showed that Lovocells transfected with the miR-222 inhibitor (800 nM) had lower invasive and migratory potentials than the control (inhibitor control). (C)The statistical results,*P<0.05. Data represent the mean ± SD of four independent experiments. (D) A microscopic image of crystal violet staining.

**Additional file 4: Figure S3.** miR-222 influence on the migration and invasion of CRC cell lines.The percent migration and invasion was calculated as the absorbance of samples/absorbance of controls×100.(A) The influence of overexpression of miR-222 on the percent of HCT8 cells that migrated and invaded (n=4). (B) miR-222 inhibitor influence on the percent of HCT8 cells that migrated and invaded(n=4). (C) The influence of overexpression of miR-222 on the percent of Lovo cells that migrated and invaded (n=4). (D) miR-222 inhibitor influence on the percent of Lovo cells that migrated and invaded(n=4).

**Additional file 5: Figure S4.** MIA3 influence on the migration and invasion of CRC cell lines.The percent of migration and invasion was calculated as the absorbance of samples/absorbance of controls×100.(A) MIA3 Inhibitor influence on the migration and invasion percent of HCT8 cells (n=4).(B) MIA3 Inhibitor influence on the migration and invasion percent of Lovo cells (n=4).

**Additional file 6: Figure S5.** The expression of MIA3 and miR-222 in CRC cell lines. (A) Western blot assay showing the expression of MIA3 protein in CRC cell lines. (B) RT-PCR assay showing the expression of miR-222 in CRC cell lines.

## Abbreviations

CRC: colorectal cancer
FCS: fetal calf serum
MIA3: melanoma inhibitory activity member 3
qRT-PCR: quantitative reverse transcription polymerase chain reaction
WT: wild type
MT: mutant type
TANGO1: transport and Golgi organization genes
