# Supplementary material for: MicroRNA-222 influences migration and invasion through MIA3 in colorectal cancer
Source: Cancer Cell Int. 2017 Aug 29;17:78. doi: 10.1186/s12935-017-0447-1 (PMC5576312; doi:10.1186/s12935-017-0447-1)
Supplement: Supplementary file 1 — Additional file 1: Table S1. The sequence of target gene (MIA33’UTR) and mutation target gene. Table S2. miR-222 had no effect on HCT8 cell cycle. Table S3. The condition of CRC patients. Table S4. The correction between miR-222 and MIA3. [file 12935_2017_447_MOESM1_ESM.docx]

Table S1 The sequence of target gene (MIA33’UTR) and mutation target gene.

| Target gene  (MIA3) | F | 5'TCGAGAATGTGCAATAAAGAATACCTGTGTTTTAGCTAATGTAGCATATGTAATTGCAAAATGATTTAGAATGTCATGAAAAGC3' |
| --- | --- | --- |
|  | R | 5'GGCCGCTTTTCATGACATTCTAAATCATTTTGCAATTACATATGCTACATTAGCTAAAACACAGGTATTCTTTATTGCACATT C3' |
| MIA3  mutation | F | 5'TCGAGAATGTGCAATAAAGAATACCTGTGTTTTAGCTAAAATTGCAAAATGATTTAGAATGTCATGAAAAGC3' |
|  | R | 5'GGCCGCTTTTCATGACATTCTAAATCATTTTGCAATTTTAGCTAAAACACAGGTATTCTTTATTGCACATT C3' |

Table S2 miR-222 had no effect on HCT8 cell cycle

|  | Mimic control | miR-222 mimic | Inhibitor control | miR-222 inhibitor |
| --- | --- | --- | --- | --- |
| G0/G1 | 43.33±0.82 | 42.4±1.75 | 43.06±1.81 | 44.66±3.68 |
| G2/M | 11.4±0.43 | 10.8±0.4 | 12.53±1.44 | 11.13±0.61 |
| S | 45.26±0.94 | 46.83±1.37 | 44.76±1.35 | 44.26±3.12 |

Table S3 The condition of CRC patients

| Number | Stage | site | Histology | Chemotherapy | Recurrence | DFS(m) | miR-222  (ΔΔCT) | miR-222  （grade） | MIA3 |
| --- | --- | --- | --- | --- | --- | --- | --- | --- | --- |
| 1 | II | colon | Adenocarcinoma | FOLFOX | 1 | 36 | 4.7 | 2 | 0 |
| 2 | II | colon | Adenocarcinoma | FOLFOX | 1 | 9 | 5.09 | 2 | 0 |
| 3 | II | colon | Adenocarcinoma | FOLFOX | 1 | 26 | 5.19 | 2 | 0 |
| 4 | II | colon | Adenocarcinoma | FOLFOX | 1 | 21 | 4.92 | 2 | 1 |
| 5 | II | colon | Adenocarcinoma | FOLFOX | 1 | 9 | 7.03 | 2 | 0 |
| 6 | III | colon | Adenocarcinoma | FOLFOX | 1 | 23 | 3.18 | 1 | 0 |
| 7 | III | colon | Adenocarcinoma | FOLFOX | 1 | 36 | 2.86 | 1 | 0 |
| 8 | III | colon | Adenocarcinoma | FOLFOX | 1 | 10 | 2.48 | 1 | 0 |
| 9 | III | colon | Adenocarcinoma | FOLFOX | 1 | 10 | 3.43 | 1 | 0 |
| 10 | III | colon | Adenocarcinoma | FOLFOX | 1 | 7 | 4.1 | 1 | 0 |
| 11 | III | colon | Adenocarcinoma | FOLFOX | 1 | 24 | 5.29 | 2 | 0 |
| 12 | III | colon | Adenocarcinoma | FOLFOX | 1 | 48 | 5.65 | 2 | 1 |
| 13 | III | colon | Adenocarcinoma | FOLFOX | 1 | 14 | 3.94 | 1 | 0 |
| 14 | III | colon | Adenocarcinoma | FOLFOX | 1 | 8 | 5.09 | 2 | 0 |
| 15 | III | colon | Adenocarcinoma | FOLFOX | 1 | 7 | 4.15 | 1 | 0 |
| 16 | III | colon | Adenocarcinoma | XELOX | 1 | 14 | 5.5 | 2 | 1 |
| 17 | III | colon | Adenocarcinoma | XELOX | 1 | 6 | 5.4 | 2 | 0 |
| 18 | III | colon | Adenocarcinoma | XELOX | 1 | 5 | 5.12 | 2 | 0 |
| 19 | III | colon | Adenocarcinoma | XELOX | 1 | 12 | 5.81 | 2 | 0 |
| 20 | II | colon | Adenocarcinoma | FOLFOX | 0 | 35 | 3.1 | 1 | 0 |
| 21 | II | colon | Adenocarcinoma | FOLFOX | 0 | 61 | 5.85 | 2 | 0 |
| 22 | II | colon | Adenocarcinoma | FOLFOX | 0 | 52 | 4.33 | 1 | 0 |
| 23 | ii | colon | Adenocarcinoma | FOLFOX | 0 | 41 | 5.63 | 2 | 1 |
| 24 | II | colon | Adenocarcinoma | FOLFOX | 0 | 52 | 5.62 | 2 | 1 |
| 25 | III | colon | Adenocarcinoma | FOLFOX | 0 | 96 | 3.37 | 1 | 0 |
| 26 | III | colon | Adenocarcinoma | FOLFOX | 0 | 72 | 3.6 | 1 | 0 |
| 27 | III | colon | Adenocarcinoma | FOLFOX | 0 | 61 | 4.11 | 1 | 0 |
| 28 | III | colon | Adenocarcinoma | FOLFOX | 0 | 38 | 3.43 | 1 | 0 |
| 29 | III | colon | Adenocarcinoma | FOLFOX | 0 | 55 | 3.58 | 1 | 0 |
| 30 | III | colon | Mucinous adenocarcinoma | FOLFOX | 0 | 59 | 5.3 | 2 | 1 |
| 31 | III | colon | Adenocarcinoma | FOLFOX | 0 | 84 | 5.14 | 2 | 1 |
| 32 | III | colon | Adenocarcinoma | FOLFOX | 0 | 41 | 5.47 | 2 | 1 |
| 33 | III | colon | Adenocarcinoma | FOLFOX | 0 | 69 | 4.24 | 1 | 0 |
| 34 | III | colon | Adenocarcinoma | FOLFOX | 0 | 40 | 4.11 | 1 | 0 |
| 35 | III | colon | Adenocarcinoma | FOLFOX | 0 | 78 | 3.57 | 1 | 0 |
| 36 | III | colon | Adenocarcinoma | XELOX | 0 | 23 | 4.27 | 1 | 0 |
| 37 | III | colon | Adenocarcinoma | XELOX | 0 | 25 | 7.29 | 2 | 1 |
| 38 | III | colon | Adenocarcinoma | XELOX | 0 | 106 | 3.64 | 1 | 0 |
| 39 | III | colon | Adenocarcinoma | XELOX | 0 | 52 | 4.46 | 1 | 0 |

Recurrence (1=recurrence; 0= no recurrence)

miR-222 was detected by RT-PCR ,the number is ΔΔCT (ΔmiR-222-Δu6)

miR-222(grade) 1=miR-222 expression Low (ΔΔCT>4.59) 2=miR-222 expression High (ΔΔCT<4.59)

MIA3 was detected by IHC. 0=negative,1=postitive.

Table S4 The correction between miR-222 and MIA3

| **Correlations** | | | |
| --- | --- | --- | --- |
|  | | miR-222 | MIA3 |
| miR-222 | Pearson Correlation | 1 | .510^**^ |
|  | Sig. (2-tailed) |  | .001 |
|  | N | 40 | 40 |
| MIA3 | Pearson Correlation | .510^**^ | 1 |
|  | Sig. (2-tailed) | .001 |  |
|  | N | 40 | 40 |
| **. Correlation is significant at the 0.01 level (2-tailed). | | | |
