# Supplementary figures and images for: MicroRNA-222 influences migration and invasion through MIA3 in colorectal cancer
Source: Cancer Cell Int. 2017 Aug 29;17:78. doi: 10.1186/s12935-017-0447-1 (PMC5576312; doi:10.1186/s12935-017-0447-1)

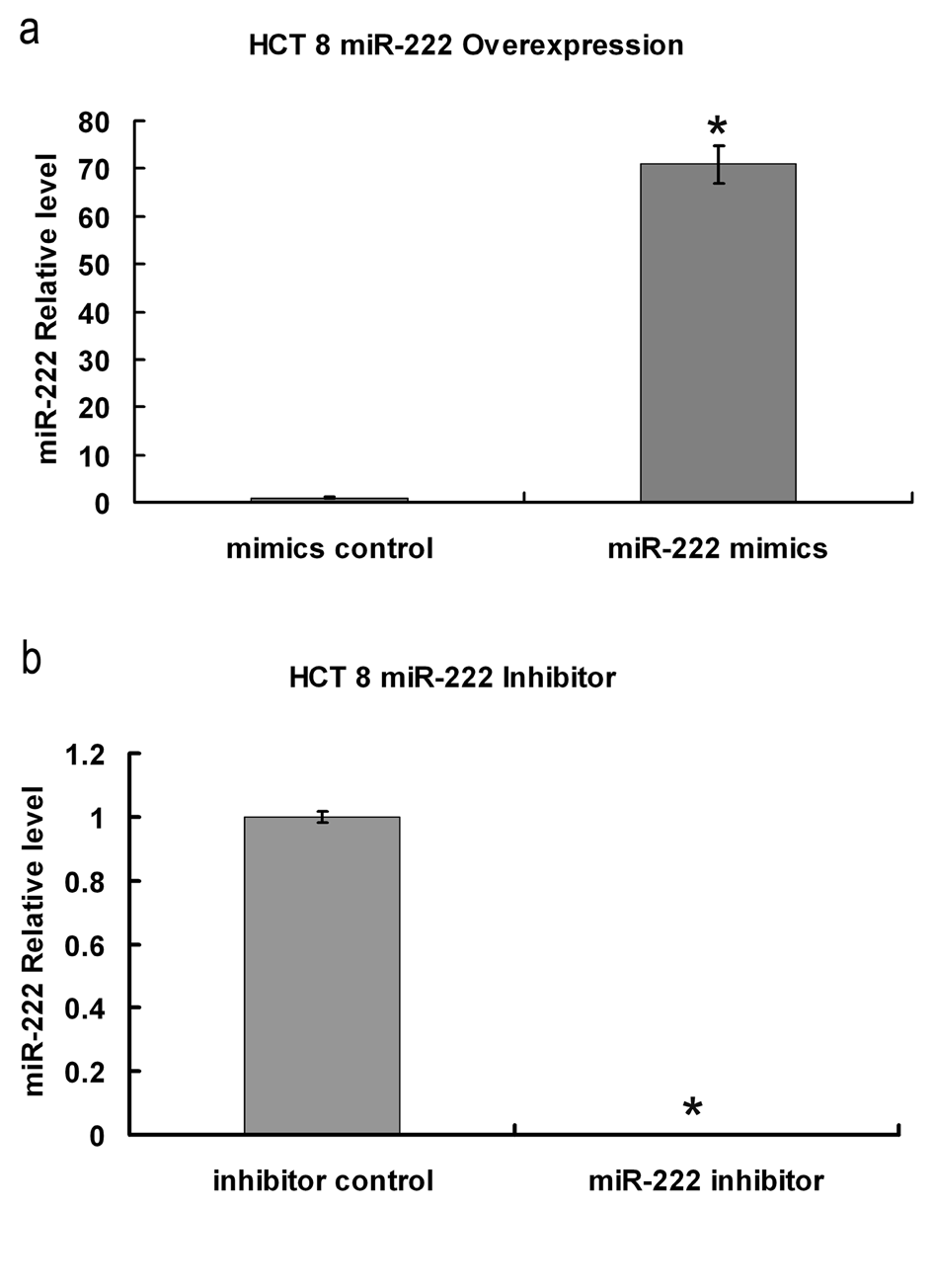

Supplement: Supplementary file 2 — Additional file 2: Figure S1. The transfection efficiency of miR-222 inhibitor and miR-222 mimics in HCT8. [file 12935_2017_447_MOESM2_ESM.tif]

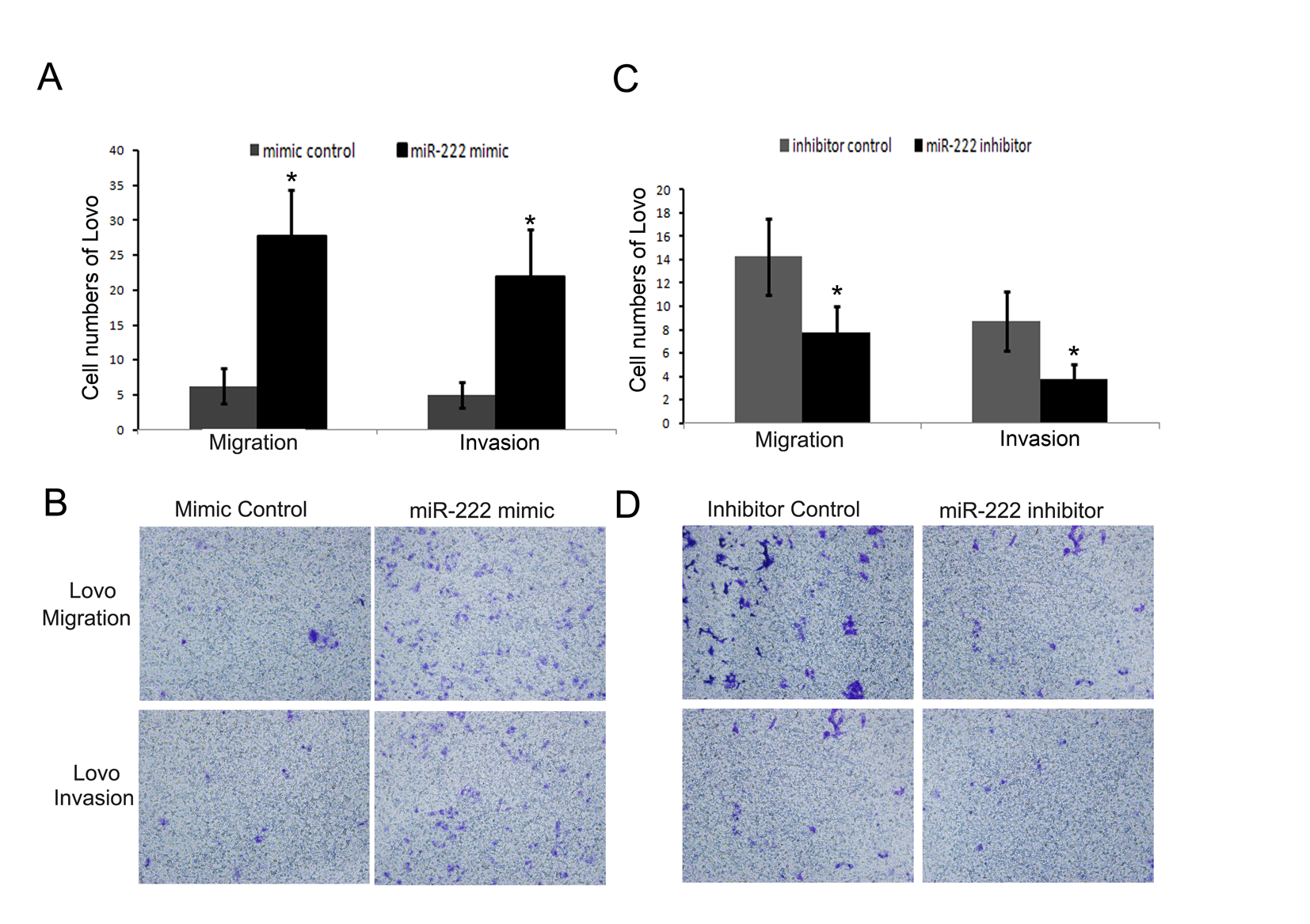

Supplement: Supplementary file 3 — Additional file 3: Figure S2. miR-222 influence on migration and invasion of Lovo. (A and B) Transwell migration (n=4) and invasion (n=4) assays showing that Lovo cells transfected with the miR-222 mimics (800 nM) had higher invasive and migratory potentials than the control (mimics control). (A) The statistical results,*P<0.05. (B) A microscopic image of crystal violet staining. (C and D) Transwell migration (n=4) and invasion (n=4) assays showed that Lovocells transfected with the miR-222 inhibitor (800 nM) had lower invasive and migratory potentials than the control (inhibitor control). (C)The statistical results,*P<0.05. Data represent the mean ± SD of four independent experiments. (D) A microscopic image of crystal violet staining. [file 12935_2017_447_MOESM3_ESM.tif]

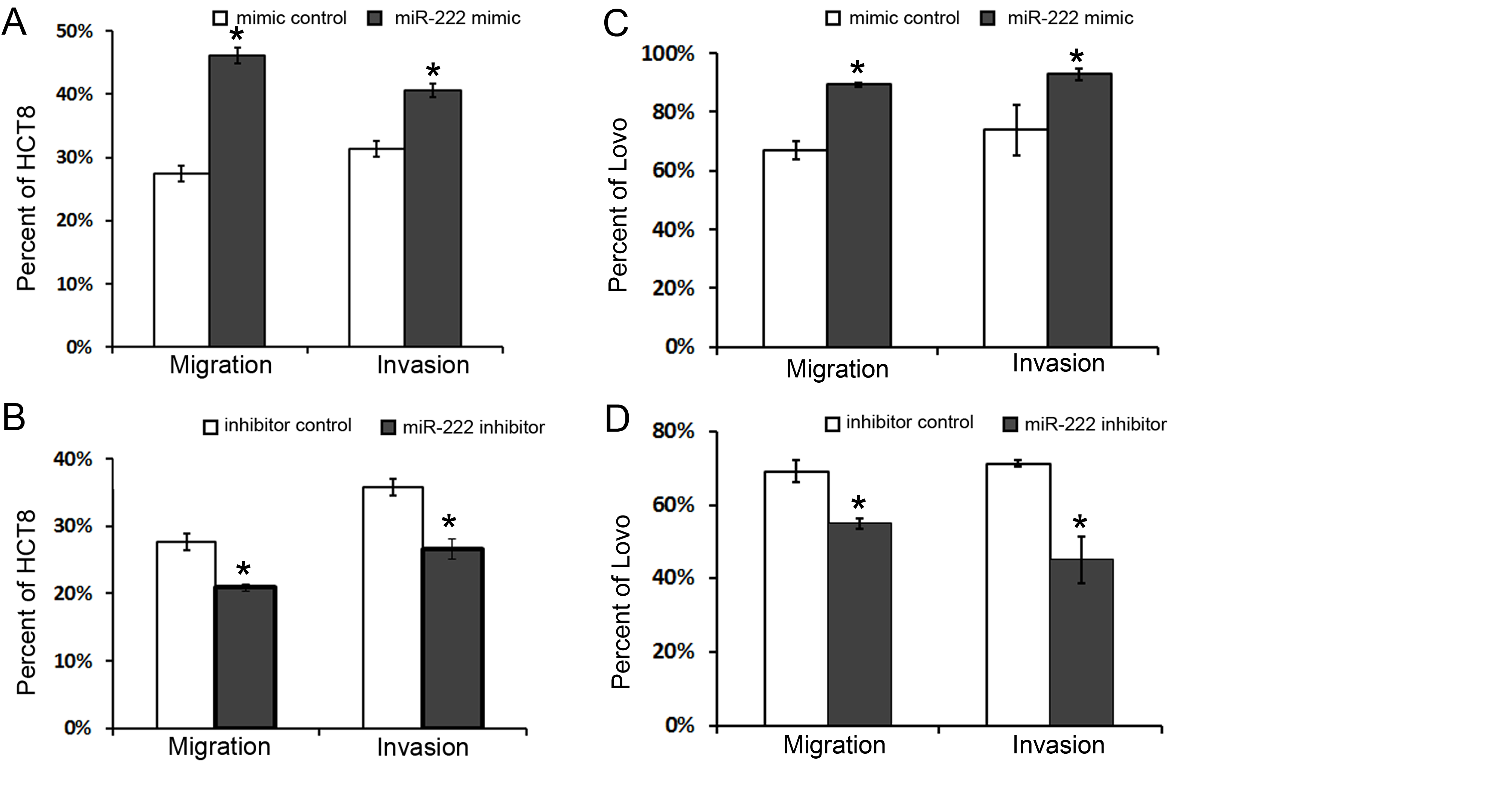

Supplement: Supplementary file 4 — Additional file 4: Figure S3. miR-222 influence on the migration and invasion of CRC cell lines.The percent migration and invasion was calculated as the absorbance of samples/absorbance of controls×100.(A) The influence of overexpression of miR-222 on the percent of HCT8 cells that migrated and invaded (n=4). (B) miR-222 inhibitor influence on the percent of HCT8 cells that migrated and invaded(n=4). (C) The influence of overexpression of miR-222 on the percent of Lovo cells that migrated and invaded (n=4). (D) miR-222 inhibitor influence on the percent of Lovo cells that migrated and invaded(n=4). [file 12935_2017_447_MOESM4_ESM.tif]

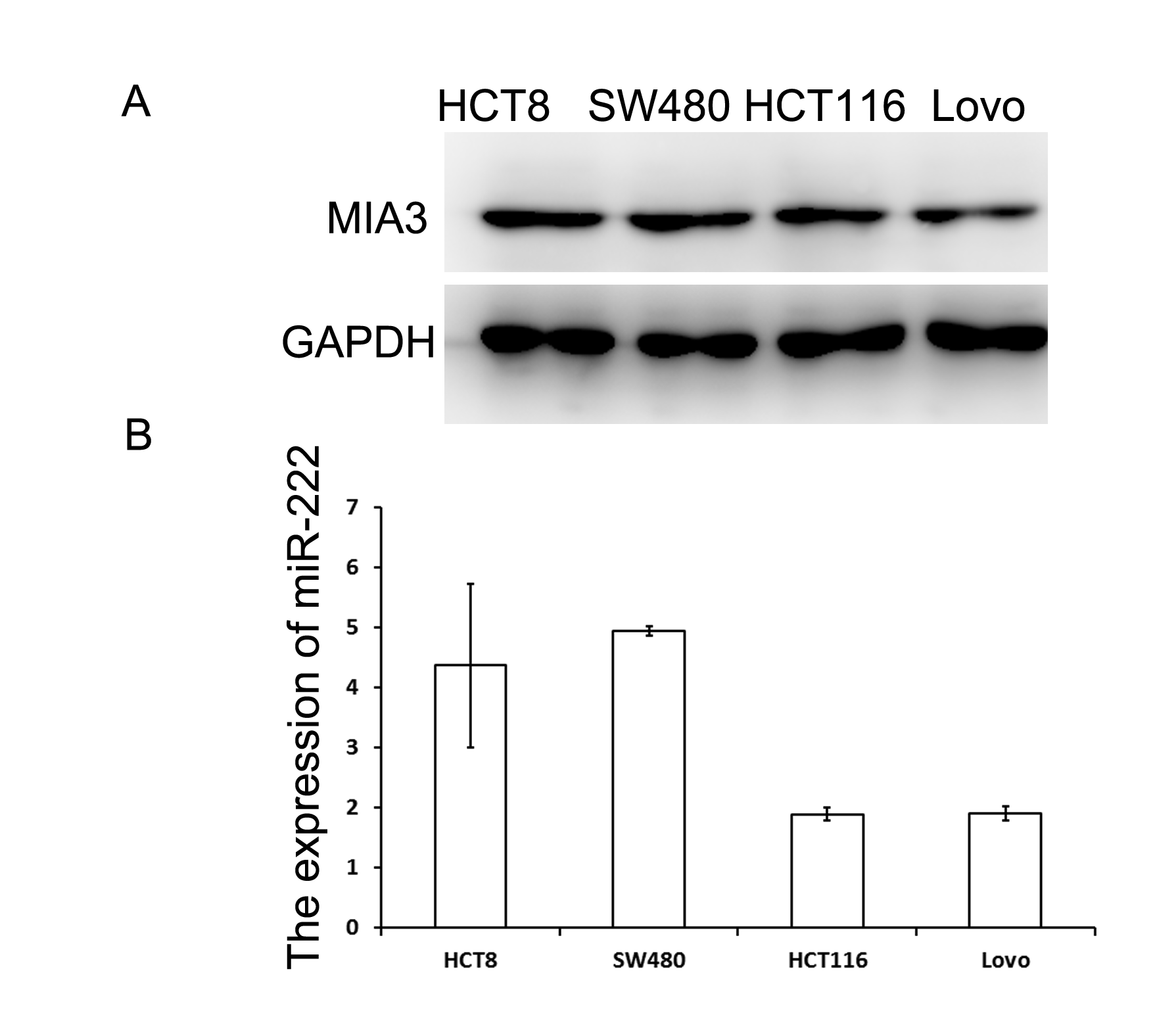

Supplement: Supplementary file 6 — Additional file 6: Figure S5. The expression of MIA3 and miR-222 in CRC cell lines. (A) Western blot assay showing the expression of MIA3 protein in CRC cell lines. (B) RT-PCR assay showing the expression of miR-222 in CRC cell lines. [file 12935_2017_447_MOESM6_ESM.tif]
